# Supplementary material for: A protocol for a systematic review of electronic early warning/track-and-trigger systems (EW/TTS) to predict clinical deterioration: Focus on automated features, technologies, and algorithms
Source: PLoS One. 2023 Mar 15;18(3):e0283010. doi: 10.1371/journal.pone.0283010 (PMC10016632; doi:10.1371/journal.pone.0283010)
Supplement: Attachment — Submitted filename: Response_to_Reviewers.docx [file pone.0283010.s002.docx]

### Rebuttal Letter

### Dear Editor of PLOS ONE

Thanks so much for the valuable comments on the manuscript (PONE-D-22-10855) entitled '' **A protocol for a systematic review of early warning/track-and-trigger systems (EW/TTS) to predict clinical deterioration: Focus on automated features, technologies, and algorithms**'' provided by reviewers. On behalf of the team of authors, I am writing the response to those comments which need reaction and correction. The changes are explained here in **blue** and presented as track changes in the text of the manuscript too. Furthermore, the revised clean version without track changes or highlights is submitted.

**Editor's comments:**

1. The abstract is too short and it looks like the expanded title. Methodology, data management, and conclusions can be included.

**The abstract is extended with further necessary information.**

2. In the Introduction, the necessity of this study is not clearly explained.

**The necessity of the study is added in the introduction section.**

3. In methodology, is there any significance in using the suggested Search Strategy?

**The reason to use the search strategy was added in the Methodology section. The sample for Scopus is added too.**

4. Is there any significance in excluding Review articles and conference papers?

**Review articles and conference papers were added to the inclusion criteria. They may have useful information for our study. Thanks**

5. PICO strategy is used in this study, but justification is not provided.

**The PICO framework is removed as there is no clinical question based on an intervention here. However, other sections cover the required information regarding the EW/TTS systems, the criteria to compare them, and the possible outcomes considered.**

6. Words such as Expert system, and prediction could be added to the search strategy.

**They were added in the related part of the search strategy.**

7. Technologies and algorithms are not discussed in this paper.

**They are discussed in the discussion section, Thanks for the comment.**

8. manuscript would be incomplete without discussing protocol efficiency.

**The discussion part was improved, thanks for the comment.**

9. Though it is a study protocol, challenges and limitations could also be presented.

**challenges and limitations were added in the discussion part.**

**Reviews' Comments :**
1. Does the manuscript provide a valid rationale for the proposed study, with clearly identified and justified research questions?
The research question outlined is expected to address a valid academic problem or topic and contribute to the base of knowledge in the field.

Reviewer #1: No

Reviewer #2: No

**The rationale for the proposed study was added in the introduction. Also, the academic problem that** **will be answered is added more clearly at the end of the introduction.**

2. Is the protocol technically sound and planned in a manner that will lead to a meaningful outcome and allow testing of the stated hypotheses?

The manuscript should describe the methods in sufficient detail to prevent undisclosed flexibility in the experimental procedure or analysis pipeline, including sufficient outcome-neutral conditions (e.g. necessary controls, absence of floor or ceiling effects) to test the proposed hypotheses and a statistical power analysis where applicable. As there may be aspects of the methodology and analysis that can only be refined once the work is undertaken, authors should outline potential assumptions and explicitly describe what aspects of the proposed analyses, if any, are exploratory.

Reviewer #1: No

Reviewer #2: No

**The methodology section is extended and more details are added.**

3. Is the methodology feasible and described in sufficient detail to allow the work to be replicable?

Descriptions of methods and materials in the protocol should be reported in sufficient detail for another researcher to reproduce all experiments and analyses. The protocol should describe the appropriate controls, sample size calculations, and replication needed to ensure that the data are robust and reproducible.

Reviewer #1: No

Reviewer #1: No

**The methodology section is extended and more details are added.**

4. Have the authors described where all data underlying the findings will be made available when the study is complete?

The [PLOS Data policy](https://journals.plos.org/plosone/s/materials-and-software-sharing) requires authors to make all data underlying the findings described in their manuscript fully available without restriction, with rare exceptions, at the time of publication. The data should be provided as part of the manuscript or its supporting information, or deposited in a public repository. For example, in addition to summary statistics, the data points behind means, medians, and variance measures should be available. If there are restrictions on publicly sharing data—e.g. participant privacy or use of data from a third party—those must be specified.

Reviewer #1: No

Reviewer #2: Yes

**We accept to provide the data available after completing the study according to the journal policy that will publish the final report. It is noted in the protocol too.**

5. Is the manuscript presented in an intelligible fashion and written in standard English?

PLOS ONE does not copyedit accepted manuscripts, so the language in submitted articles must be clear, correct, and unambiguous. Any typographical or grammatical errors should be corrected at revision, so please note any specific errors here.

Reviewer #1: Yes

Reviewer #2: No

**The whole manuscript was checked for errors and corrections.**

**Review Comments to the Author**

Reviewer #1: I am unable to understand the significance of this work in its current form.
The authors have merely described some plan of action for performing a review on the prediction of clinical deterioration. Rather, it will be certainly interesting if the review has been actually performed and the findings are discussed in detail.

Reviewer #2: A review to predict clinical deterioration is performed using PRISMA. Following are a few questions for the authors:
1. CD is a wide term; authors can try to be more specific.

**A more specific definition is provided in the introduction.**

2. Authors have presented a few results/observations from earlier publications. It is not clear what critical conclusions arrive.

**The introduction is improved and a clear conclusion from the review of studies was added.**

3. Authors have used different terms like features, AI, IoT, etc. But no clear and in-depth discussions are found. What is the inference drawn from this? How will it be useful for future scientists/researchers?

**The discussion section is added with details regarding the technologies, limitations, challenges, and future works.**
