# Supplementary material for: A protocol for a systematic review of electronic early warning/track-and-trigger systems (EW/TTS) to predict clinical deterioration: Focus on automated features, technologies, and algorithms
Source: PLoS One. 2023 Mar 15;18(3):e0283010. doi: 10.1371/journal.pone.0283010 (PMC10016632; doi:10.1371/journal.pone.0283010)
Supplement: Attachment — Submitted filename: Rebuttal Letter_round_2.docx [file pone.0283010.s003.docx]

Rebuttal Letter

Dear Editor of PLOS ONE

Thanks so much for the valuable comments on the manuscript (PONE-D-22-10855R1) entitled ''A protocol for a systematic review of early warning/track-and-trigger systems (EW/TTS) to predict clinical deterioration: Focus on automated features, technologies, and algorithms'' provided by reviewers. On behalf of the team of authors, I am writing the response to those comments which need reaction and correction. The changes are explained here in **blue** and presented as track changes in the text of the manuscript too. Furthermore, the revised clean version without track changes or highlights is submitted.

Journal Requirements :
1. Please review your reference list to ensure that it is complete and correct. If you have cited papers that have been retracted, please include the rationale for doing so in the manuscript text, or remove these references and replace them with relevant current references. Any changes to the reference list should be mentioned in the rebuttal letter that accompanies your revised manuscript. If you need to cite a retracted article, indicate the article’s retracted status in the References list and also include a citation and full reference for the retraction notice.

All references were checked. Here there are the changes for the references:

The references 9 and 10 were replaced with each other. But still, we have both of them in the references.

References 21, 22, 23, 24, and 34 were added.

2. Statistical analysis of the outcome or any recommendation to future researchers is not elaborated.

The statistical analysis of the outcome and further studies suggestions were added in the discussion section.

Reviewers' comments:
1. Does the manuscript provide a valid rationale for the proposed study, with clearly identified and justified research questions?

The research question outlined is expected to address a valid academic problem or topic and contribute to the base of knowledge in the field.

Reviewer #1: Yes

Reviewer #2: No

The introduction section is revised to improve the study justifications. Also, in the discussion section, we added the provided knowledge by this study which can be the basis for further studies and system developments.

2. Is the protocol technically sound and planned in a manner that will lead to a meaningful outcome and allow testing the stated hypotheses?

The manuscript should describe the methods in sufficient detail to prevent undisclosed flexibility in the experimental procedure or analysis pipeline, including sufficient outcome-neutral conditions (e.g. necessary controls, absence of floor or ceiling effects) to test the proposed hypotheses and a statistical power analysis where applicable. As there may be aspects of the methodology and analysis which can only be refined once the work is undertaken, authors should outline potential assumptions and explicitly describe what aspects of the proposed analyses, if any, are exploratory.

Reviewer #1: Yes

Reviewer #2: Yes

3. Is the methodology feasible and described in sufficient detail to allow the work to be replicable?


Descriptions of methods and materials in the protocol should be reported in sufficient detail for another researcher to reproduce all experiments and analyses. The protocol should describe the appropriate controls, sample size calculations, and replication needed to ensure that the data are robust and reproducible.

Reviewer #1: Yes

Reviewer #2: Yes

4. Have the authors described where all data underlying the findings will be made available when the study is complete?

The [PLOS Data policy](https://journals.plos.org/plosone/s/materials-and-software-sharing) requires authors to make all data underlying the findings described in their manuscript fully available without restriction, with rare exception, at the time of publication. The data should be provided as part of the manuscript or its supporting information, or deposited to a public repository. For example, in addition to summary statistics, the data points behind means, medians and variance measures should be available. If there are restrictions on publicly sharing data—e.g. participant privacy or use of data from a third party—those must be specified.

Reviewer #1: Yes

Reviewer #2: Yes

5. Is the manuscript presented in an intelligible fashion and written in standard English?

PLOS ONE does not copyedit accepted manuscripts, so the language in submitted articles must be clear, correct, and unambiguous. Any typographical or grammatical errors should be corrected at revision, so please note any specific errors here.

Reviewer #1: Yes

Reviewer #2: Yes

6. Review Comments to the Author

Please use the space provided to explain your answers to the questions above and, if applicable, provide comments about issues authors must address before this protocol can be accepted for publication. You may also include additional comments for the author, including concerns about research or publication ethics.

Reviewer #1: The authors have implemented all the suggestions provided. The manuscript has been now modified with all the required details.

Reviewer #2: Yes authors have addressed a few of the reviewer's comments. The major portion of the paper talks about the general introduction and review process. Statistical analysis of the outcome or any recommendation to future researchers is not elaborated.

The required improvements were applied in the introduction and discussion sections.

- Few grammatical/typos can be looked into carefully.

The whole paper was double-checked for grammatical/typos. Thanks for the comment.

**The manuscript is really improved.**

**Thanks so much for the constructive comments.**
