# Supplementary material for: A protocol for a systematic review of electronic early warning/track-and-trigger systems (EW/TTS) to predict clinical deterioration: Focus on automated features, technologies, and algorithms
Source: PLoS One. 2023 Mar 15;18(3):e0283010. doi: 10.1371/journal.pone.0283010 (PMC10016632; doi:10.1371/journal.pone.0283010)
Supplement: Attachment — Submitted filename: Rebuttal Letter_round_3.docx [file pone.0283010.s004.docx]

Rebuttal Letter

Dear Editor of PLOS ONE

Thanks so much for the valuable comments on the manuscript (PONE-D-22-10855R1) entitled ''A protocol for a systematic review of early warning/track-and-trigger systems (EW/TTS) to predict clinical deterioration: Focus on automated features, technologies, and algorithms'' provided by reviewers. On behalf of the team of authors, I am writing the response to those comments which need reaction and correction. The changes are explained here in **blue** and presented as track changes in the text of the manuscript too. Furthermore, the revised clean version without track changes or highlights is submitted.

Journal Requirements:

Please review your reference list to ensure that it is complete and correct. If you have cited papers that have been retracted, please include the rationale for doing so in the manuscript text, or remove these references and replace them with relevant current references. Any changes to the reference list should be mentioned in the rebuttal letter that accompanies your revised manuscript. If you need to cite a retracted article, indicate the article’s retracted status in the References list and also include a citation and full reference for the retraction notice.

**The whole references were rechecked and sorted based on the Journals format.**

Additional Editor Comments:

1. Statistical analysis part needs to be explained more for better understandability of readers.

**It is written with more explanation in more understandable version.**

2. Language can be further improved.

**The whole doc was rechecked and the text was improved.**

3. Referencing style can be formatted as per journal requirements.

**It is done.**

**Thanks very much.**
